# Less-advanced regions in EU innovation networks: Could nanotechnology represent a possible trigger for path upgrading?

**DOI:** 10.1371/journal.pone.0288669

**Published:** 2024-01-12

**Authors:** Giuseppe Calignano, Anne Jørgensen Nordli

**Affiliations:** 1 Inland School of Business and Social Sciences, Department of Organisation, Leadership and Management, Inland Norway University of Applied Sciences, Lillehammer, Norway; 2 Inland School of Business and Social Sciences, Department of Business Administration, Inland Norway University of Applied Sciences, Lillehammer, Norway; University of Economics Ho Chi Minh City, VIET NAM

## Abstract

This paper examines whether nanotechnology projects funded under the European Union (EU) Framework Programmes (FPs) are a possible trigger for path upgrading (i.e., infusion of new technologies in existing traditional sectors) in less-advanced regions. First, the adoption of cluster analysis and a set of key indicators (i.e., technological intensity, scientific excellence, human capital, and research and development expenditure) allowed us to distinguish between 79 more-advanced and 127 less-advanced EU regions. Subsequently, through social network analysis and nonparametric testing we were able to demonstrate how the less-advanced EU regions (average degree centrality: 40.5) play a marginal role compared with the more-advanced ones (average degree centrality: 98.5) in the nanotechnology network created within Horizon 2020—i.e., the EU programming cycle implemented in the 2014–2020 period. Despite this, we observed that a few less-advanced regions (33 out of 127) were able to score higher than the EU median in terms of participation in the targeted nanotechnology network, thus benefiting from relevant knowledge flows potentially leading to re-industrialization processes. The adoption of qualitative comparative analysis allowed us to determine which combinations of key innovation, scientific and socioeconomic factors could facilitate such beneficial interregional interactions and related knowledge exchange in these types of regions (i.e., primarily what we defined as “relative innovativeness,” excellence in nanotechnology research and a comparatively high level of gross domestic product per capita). Our empirical results provided some clear policy implications. For instance, the necessity to I) remove the barriers impeding a more balanced participation to promote a widespread renewal of traditional industries in less-advanced regions and II) implement coordinated EU and domestic actions designed to encourage the involvement of the great majority of the less-advanced regions, which remain marginal in the periodically launched FPs.

## 1. Introduction

Nanotechnology is a disruptive interdisciplinary field by virtue of its potential application in a multitude of both high-technology and science-based sectors (biotechnology, cognitive science, information and communication technology, etc.) and traditional industries (textile, ceramics, food, etc.) [1]. Its multidisciplinary dimension has led the European Union (EU) to include nanotechnology in the key enabling technologies (KETs) that could potentially help countries and regions tackle grand societal challenges and foster advanced and sustainable national and regional economies by means of new jobs and economic growth [2]. In particular, the versatility of nanotechnology and the opportunity to employ it in many different medium- and low-technology sectors [1] make it the ideal medium through which to study possible “path upgrading” [3] in less-advanced regions. In this paper, we distinguish between more-advanced and less-advanced regions based on their technological intensity, scientific excellence, human capital, and research and development (R&D) expenditure (see Section 3.1 and S1 Table for how these indicators are operationalized and built).

According to Asheim et al. (2019) [4], path upgrading reflects a major change in an established industrial path in a new direction, based on the adoption of new technologies, that activates a renewal process in existing industries. Thus, this study assumes that a high level of participation or playing an active role in nanotechnology networks may be symptomatic or represent the harbinger of path upgrading in less-advanced regions by virtue of their ability to acquire relevant knowledge and technology through cooperation in transnational innovation networks. This seems to be facilitated by an analytical knowledge base that mainly characterizes science-based sectors such as nanotechnology because knowledge is primarily transferred through codified channels (e.g., co-publications and patenting), which in principle do not represent an obstacle to effective interaction with geographically distant partners (see Asheim and Gertler, 2005) [5].

In this regard, recent work on new development paths unequivocally suggests that relatively less-developed innovation systems (i.e., “thin” and “thick and specialized”; see [3, 6]) are poorly equipped to support the emergence of completely new economic activities, but may have the capacity to facilitate the upgrading of old industries [3, 4, 7]). In other words, what we argue is that if “thinner” or less-advanced regions show a certain ability to benefit from the knowledge flows engendered by multiscalar collaborations, this may lead to a beneficial infusion of new technologies in existing obsolete technological paths. In a similar vein, Calignano and Quarta [8] argues that new technologies that penetrate and catalyze innovation activities in traditional sectors may trigger a process of “re-industrialization” in peripheral and less-innovative regional areas.

Based on these brief considerations, and by adopting a relational multiscalar approach to the study of regional development (e.g., [9, 10]), this paper examines whether the nanotechnology projects funded by the EU under the Horizon 2020 programme (hereinafter H2020-Nanotech) could be one possible way to trigger path upgrading [11] or re-industrialization [8] in less-advanced regions. This was done by investigating the capacity shown by such a specific typology of EU regions to be embedded into the transnational nanotechnology network under analysis. Moreover, another objective of this paper is to show the combination of structural properties (i.e., relative innovativeness, excellence in nanotechnology research, high level of non-R&D innovation expenditure, size of the economy, quality of government and resident population) that enables a high level of participation of the less-advanced regions in research and innovation networks such as the ones fostered by the EU.

The aim of H2020—which is the eighth EU FP—was to fund competitive consortia and highly innovative joint research projects in given topics or fields (including nanotechnology). With specific regard to the objective of this paper, it is useful to highlight how the EU has constantly and explicitly adopted a relational and multiscalar approach in its innovation policy (i.e., funds allocated to competitive collaborative projects involving partners from different geographical areas; [12]). In this regard, it is also interesting to note that, among other things and throughout the latest FP (2014–2020 period), the EU implemented specific policy actions to enhance the number of applicants and the related participation of the regional organizations located in less-developed regions and countries [13]. This conveys the idea of how more balanced participation between more- and less-advanced regions represents a primary concern for the EU.

In this paper, we have used various statistical sources (Community Research and Development Information Service (CORDIS), Eurostat, other EU databases, etc.) and techniques (social network analysis (SNA), cluster analysis and nonparametric tests) to determine whether the joint nanotechnology projects funded under the last concluded EU FP are an effective means of triggering path upgrading in less-advanced EU regions. In addition, we adopted crisp-set qualitative comparative analysis (csQCA) to determine what factors could allow less-advanced EU regions to score highly in terms of network centrality in the H2020-Nanotech programme, thus potentially enabling beneficial re-industrialization processes.

This paper contributes to the extant literature in several ways, e.g., by providing research, methodological and policy inputs and insights. First, to our knowledge, this paper represents the first attempt to study possible path upgrading [11] by adopting a multiscalar relational approach and, above all, by using large databases and quantitative methods. In this respect, another relevant element of novelty is the adoption of an original combination of statistical techniques such as cluster analysis, SNA, nonparametric testing, and a cutting-edge and largely unexplored method in the field of economic geography known as qualitative comparative analysis (QCA). Finally, our results allowed us to reflect thoroughly on the main direct and indirect implications of our study from the policy viewpoint. Based on this, we have tried to provide useful insights for policymakers and evaluators to foster new development paths in less-advanced EU regions.

The remainder of this paper is organized as follows. In Section 2, we outline the theoretical background of our study by reviewing the main literature on the topics that we primarily tackle—i.e., nanotechnology applications and related economic and geographical implications, the definition of more- and less-advanced regions, the importance of the collaborative dimension of innovation, and the main spatial and relational characteristics of the EU research and innovation networks. In Section 3, we describe in detail the network analysis and statistical methods that we adopted to conduct the empirical analysis referring to the knowledge exchange engendered by H2020-Nanotech in less-advanced regions. In Section 4, we present the main results of our study, which we discuss in the concluding Section 5.

## 2. Literature review

In the next three subsections, we first introduce the main characteristics of nanotechnology and briefly return to the nexus between its application and possible path upgrading in marginally innovative geographical contexts. Subsequently, we clarify how we classify the targeted EU regions as more- or less-advanced, based on a simplified version of the typification of the different possible regional innovation systems (RISs) introduced by Isaksen and Trippl [3]. Finally, in the third subsection, we explain how the relational approach to the study of regional development informs the case study presented in this paper, and describe some key characteristics of the multiscalar innovation networks that have been fostered throughout the various programming cycles periodically launched by the EU. The aim of all these subsections is to give the reader a thorough understanding of the background in which our empirical analysis is embedded.

**Fig 1:**
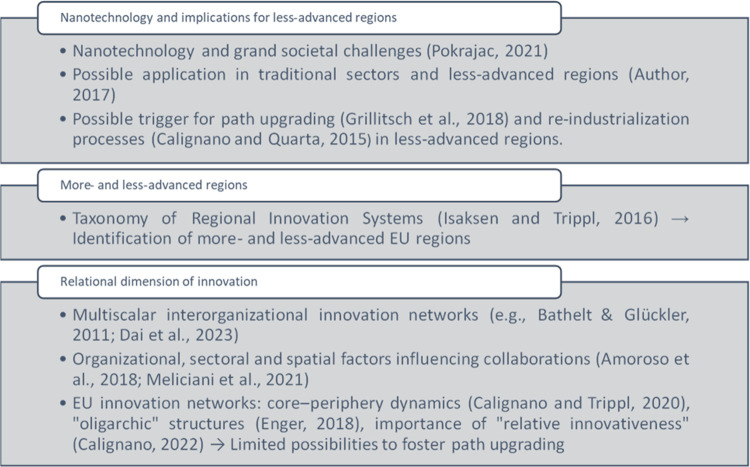
Theoretical framework: Main topics and concepts.

The various parts making up our theoretical framework are used to establish a subsequent clear nexus between our input variables, response variable and detected phenomena. As briefly mentioned above and described in detail subsequently, we argue that a high degree of participation in innovation networks such as H2020-Nanotech (i.e., our response variable or outcome in QCA terms; see Section 2.1) may lead to possible path upgrading [11] in less-advanced regions (see Section 2.2) by means of beneficial multiscalar knowledge flows (see Section 2.3). The combined adoption of SNA and csQCA allowed us to determine the uneven participation of more- and less-advanced regions, as well as under which conditions (input variables) a very few less-advanced regions show higher scores in terms of degree centrality, thus benefiting from knowledge exchange and positive network effects potentially engendering re-industrialization processes [8]. The three subsections and the related topics making up our theoretical background are illustrated graphically and systematized through SmartArt graphics in Fig 1.

### 2.1. Nanotechnology: Characteristics, applications and implications for geographical research

Effective use of resources such as energy, water, food [14–16], climate change [17–19], therapeutic solutions for an increasingly elderly population [20], and widespread and sustainable adoption of information and communication technology [21] represent only a few of the grand societal challenges that could be tackled through the adoption of nanotechnology solutions [22]. All of these pressing issues should be addressed by adopting a coordinated multidimensional and multiscalar perspective (e.g., [23]) in which systemic innovation policy plays a key role [24]. As we briefly mentioned above, this is one of the reasons why the EU has decided to prioritize nanotechnology in its research, innovation and industrial policies, which have currently a strong focus on responsible and sustainable development (e.g., [25]).

According to the International Organization for Standardization, nanotechnology can be defined as the “[u]nderstanding and control of matter and processes at the nanoscale, typically, but not exclusively, below 100 nanometres in one or more dimensions where the onset of size-dependent phenomena usually enables novel applications” [26]. These applications are innumerable because nanotechnology-enabled products and services can be incorporated into a very large number of industrial sectors and medical fields, potentially leading to increased regional productivity, job creation and more sustainable development [27]. In addition to high-technology sectors (e.g., chemicals and pharmaceuticals, aerospace, electronics, telecommunications), nanotechnology may be successfully applied in many different traditional sectors (food, textiles, sporting goods and ceramics, to mention just a few; see [1]). This phenomenon may lead to considerable positive consequences for the development paths of less-advanced regions—i.e., the geographical areas where medium-low-technology and low-technology industries are predominantly and traditionally located (e.g., [3, 28]). This is precisely what Calignano and Quarta [8] refer to when they suggest that widespread adoption of nanotechnology in traditional sectors could help less-innovative and, more generally, less-advanced regions to narrow the industrial development gap toward the most advanced players by triggering re-industrialization processes. This approach shows more than one analogy with the subcategory of path upgrading named “renewal”: “[S]uch processes […] triggered by the infusion of new technologies” that may lead to new development paths in less-developed regions ([11]; p. 265). For this reason, the terms “path upgrading” and “re-industrialization” are used interchangeably in this paper.

### 2.2. More- and less-advanced regions

Possible new development paths may be related to different types of RIS. In their initially proposed and subsequently expanded taxonomy, Isaksen and Trippl [3] distinguish between three types of RIS, based on their level of development: I) organizationally thick and diversified RISs, II) organizationally thick and specialized RISs and III) thin RISs. These three types of RIS differ predominantly in terms of their industrial structure, knowledge base and institutional set-up, with the thick and diversified RISs showing more favorable conditions for new development paths to take place by virtue of their broader industrial diversity and the presence of high-technology sectors, leading universities and research organizations [29]. Conversely, thick and specialized RISs often coincide with older regional industrial areas and are generally characterized by a narrower industrial base and specialization in traditional manufacturing sectors [30–33]. Finally, thin RISs are geographically remote areas that are characterized by weakly developed clusters, an absence of research organizations and a lack of knowledge exchange between the few regional companies [34].

Starting from the taxonomy originally proposed by Isaksen and Trippl [3], the EU regions surveyed in the empirical part of this paper were classified as more- or less-advanced, based on their relative levels of technological intensity, scientific excellence, human capital and expenditure on R&D (see Section 3 and S1 Table for details).

Our main interest concerns the role played by less-developed regions in the targeted EU nanotechnology network. Specifically, greater participation and key positioning of more-advanced regions (which largely correspond to the thick and diversified RISs, as defined by Isaksen and Trippl, [3]) are assumed to lead to further reinforcement of innovation activities in more innovative and already successful regions. Conversely, holding a central position could contribute positively to path upgrading in less-advanced EU regions (i.e., infusion of new technologies, generally in traditional sectors, which could lead to changes or new directions for the existing industrial path; [11]). In this regard, it becomes of major interest to understand the drivers behind the positioning of the less-advanced regions in H2020-Nanotech, which is a critical aspect that we examined through the adoption of QCA. Based on all of these considerations, and with the aim to provide the basis for investigating further the role of less-advanced regions in H2020-Nanotech, we formulate our first research question (RQ1) as follows: *How are the targeted EU regions clustered into more- or less-developed ones*?

### 2.3. A relational approach to the study of regional development: EU innovation networks

Relevant strands of literature in economic geography and innovation studies stress the importance of the collaborative dimension of innovation (e.g., [35–37]). Nowadays, in a complex and constantly evolving world, innovation generally takes place in interorganizational networks where various types of organizations, such as private companies and research institutions, exchange people, resources and essential knowledge [38].

These innovation networks are organized on various geographical scales (from regional to global; e.g., [9, 39]) and may be engendered and sustained by face-to-face contacts and/or interactions with more distant partners (in these latter cases, the literature on the topic stresses the importance of temporary proximity and computer-mediated communication; see e.g., [40, 41]). Although the growing importance of transnational cooperation in interactive learning processes has been demonstrated in various empirical analyses [42, 43], the geographical dynamics that determine collaboration patterns within multiscalar innovation networks depend largely on organizational, sectoral and spatial factors such as absorptive capacity, geographical distance to core regions and the knowledge bases that primarily characterize given sectors and industries (see [44–46]).

As we briefly mentioned above, this research is conducted in the context of H2020-Nanotech, which is the most recently concluded FP and was implemented by the EU in the 2014–2020 period. The FPs represent the most important EU research and innovation policy [47]. Since the launch of the first programming cycle in 1984, the EU has aimed constantly at funding the most promising innovative research projects in many different fields, with the objective of creating the best possible environment for allowing different types of actors to exchange knowledge and disseminate ideas with potential industrial applications [1]. To achieve these goals, the EU FPs explicitly adopt a multiscalar collaborative approach and promote the creation of research consortia comprising industrial and research organizations from at least three different countries [48]. One of the main objectives of the EU is to promote the achievement of well-balanced participation between the various EU regions by means of various policy recommendations and actions (e.g., the “Spreading Excellence and Widening Participation Programme”; see [49]). This result would be desirable and consistent with both the Cohesion Policy implemented by the EU (aimed at “harmonious development” between more- and less-developed countries and regions) and the strategies of the European Research Area (i.e., integrating the scientific resources, and enabling the circulation of talented people, knowledge and technologies) [13, 50]. This leads us to formulate our second research question (RQ2): *How involved are the less-developed EU regions in the nanotechnology network created under the H2020 programme*?

Despite such openly declared objectives, however, the research and innovation networks created and fostered by the EU FPs are characterized traditionally by evident core–periphery dynamics [51, 52], in which a clear and stable core of countries, regions and organizations can be identified throughout the various EU FPs [53]. In particular, the most central and developed regions tend to be more or better connected, thereby holding key positions in the network core (e.g., [54, 55]), with the less-developed regions playing only a marginal role and linking themselves primarily to core regions and countries [44, 56].

In addition to providing vital research funds for regional organizations, a high level of participation in the EU FPs may contribute to knowledge diffusion and the promotion of different development paths in different types of regions, as shown by recent studies on the topic [57–59]. In further detail, participation in strongly specialized regional areas may potentially enhance branching (diversification into related sectors), while it may foster path creation (diversification in completely new or unrelated sectors) in the more-developed regions [56]. However, what this study and similar ones (e.g., [52]) reveal is that narrower knowledge bases and capabilities may represent a hindrance to the satisfactory participation of less-advanced regions in the EU research and innovation network.

Despite the existence of “oligarchic networks” that are dominated by a few organizations and regions (see [60, 61] in this regard), a recent study conducted by Calignano [62] demonstrates how regions that show higher levels of innovativeness than the respective national average achieve satisfactory participation in the EU innovation networks, even in the case of less-advanced regions that are situated in marginally or only moderately innovative countries. This seems to be consistent with another empirical analysis that demonstrates how less-advanced regions may, in some cases, be particularly innovative and well connected to regional and more geographically distant partners [63].

Based on these previous findings, this paper examines which combination of structural and economic factors actually fosters a good number of participations in the targeted EU nanotechnology network. These possible factors include critical structural and economic aspects, such as being more innovative than the respective national median (which can be defined as “relative innovativeness”; see [62]), in addition to excellence in nanotechnology, non-R&D innovation expenditure, quality of regional government, economic size and population (see Section 3.3 for a detailed description of this specific analysis carried out by means of csQCA). This leads to our third research question (RQ3): *Which combinations of key factors allow less-developed regions of Europe to score highly and be consequently adequately engaged in the EU nanotechnology network*?

## 3. Methodological strategy

**Fig 2:**
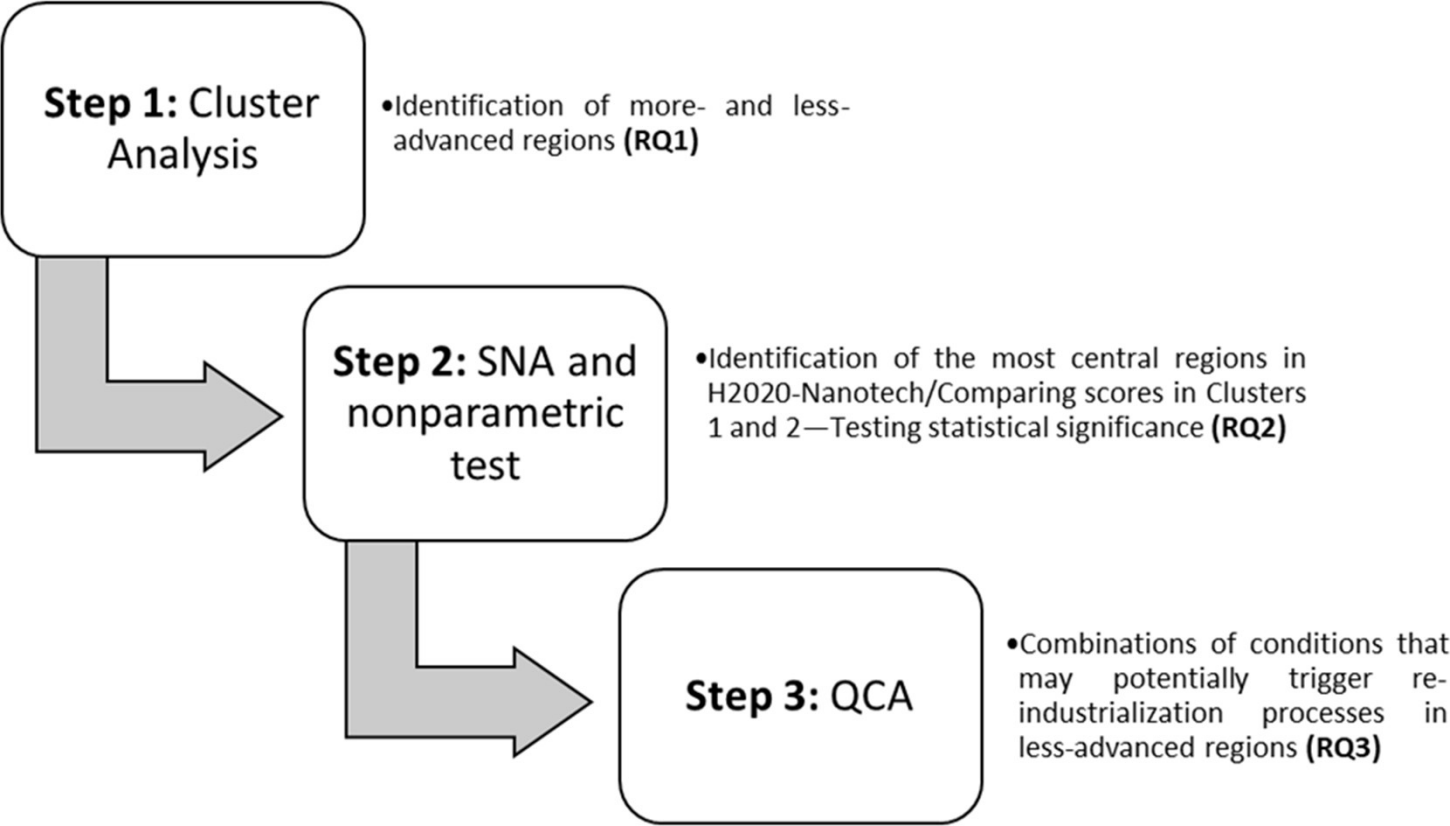
Graphical visualization of the three steps making up the methodological approach.

The above literature review represents the analytical framework of our empirical analysis, which was conducted in three steps (see Fig 2). First, we addressed RQ1 by identifying the more- and less-advanced EU regions through the adoption of cluster analysis. We then used SNA to calculate the network centrality of each region in the context of the H2020-Nanotech programme (RQ2), and we used nonparametric tests to determine whether considerable and statistically significant differences in the level of participation can be observed between more- and less-developed regions. Finally, we addressed RQ3 by conducting csQCA to identify which combinations of factors enable a high level of participation by the less-advanced regions in the aforementioned targeted nanotechnology network.

### 3.1. Step 1—Cluster analysis

In the first step, we used cluster analysis to classify the surveyed EU regions as either more- or less-advanced (NUTS 0, 1 and 2, based on how they are classified in the European and Regional Innovation Scoreboards 2014 (EIS-RIS2014), which is the edition published in the year in which H2020-Nanotech was launched). Cluster analysis is chosen because it is a particularly relevant methodology when the goal is to identify objects with the same characteristics. Specifically, we used seven variables, corresponding to four indicators, to divide the EU regions into two groups according to their level of development. These indicators were chosen based on critical elements for regional development, as identified by the main literature on RISs and related development paths (among many others, see the recent studies by [62]):

I) Technological intensity: average share of total employment in industrial sectors classified according to technological intensity and based on NACE Rev. 2 (high technology, medium-low technology and low technology);

II) Scientific excellence: scientific publications among the top 10% most cited publications worldwide as a percentage of total scientific publications of the respective country;

III) Human capital: share of the population who have completed tertiary education;

IV) R&D spending: R&D expenditure in the public and business sectors as a percentage of gross domestic product (GDP) (see S1 Table for details of the variables associated with each indicator and the databases in which they can be found).

K-means clustering is the method that we used to classify the targeted EU regions. This clustering technique allows researchers to create a given number of clusters K, in which the objects(the EU regions, in the present case study) are grouped on the basis of high intracluster and low intercluster similarity. This takes place by means of an iterative step-by-step process in which the clustering algorithm identifies various centroids (i.e., the means representing the cluster centers) until convergence is reached and the objects in the identified clusters remain stable. The main drawback of K-means clustering is that there is no clear theoretical approach to determine the final number of clusters, which is set by the individual researchers based on their subjective parameters, knowledge of the context subject to analysis and specific research needs or hypotheses to be tested. There are nonetheless certain methods that can be applied to validate the number of clusters identified—e.g., checking the iteration history and post hoc nonparametric tests—which we carried out and reported in S2 Table and S1 Fig.

### 3.2. Step 2—SNA and nonparametric tests

After determining the regions that make up Cluster 1 (more-advanced regions) and Cluster 2 (less-advanced regions), the second step involved the adoption of several SNA techniques to identify the most central regions in H2020-Nanotech and to reveal certain structural properties of the targeted network (see [64], for further specification of the SNA measures applied in this paper). In particular:

- Degree centrality was used to calculate the overall number of ties established by the regional organizations located in each EU region;

Because of the relational approach of studying regional development and our interest in networks of collaboration, SNA techniques are particularly suited to addressing our research question.

- The core–periphery model was used to identify a hypothetical densely connected “core” and a loosely connected “periphery” (see [65]), whereas the related “fitness” was calculated to indicate how well the observed data approximate such an ideal structure;
- Density by Group was used to determine the different densities (i.e., the total number of observed ties divided by the total number of possible ties) within and between the two identified clusters, thus revealing how concentrated knowledge exchange is in the case of more- and less-advanced regions.

To perform network analysis, we used a case-by-case matrix in which the surveyed EU regions represent the cases, and the collaborations between organizations located in these regions represent the ties. In particular, two regions were considered to be connected when they participated at least once in a joint research project funded as part of the H2020-Nanotech programme. We built our database in May 2020 and, overall, we surveyed 853 nanotechnology and nanoscience projects that were funded by the H2020 programme, which saw 5,485 participations by different types of organizations (firms, universities, research centers, public authorities and agencies, etc.) located in 206 EU regions.

Subsequently, we conducted a Mann–Whitney *U* test to understand whether the differences observed between more-advanced and less-advanced EU regions in terms of node centrality and key positioning in H2020-Nanotech are statistically significant and lead to reliable and informative results. The Mann–Whitney *U* is a nonparametric test that allowed us to test the zero hypothesis, according to which the distribution of degree centrality is equal for the two types of regions identified through K-means clustering and classified as more- (Cluster 1) or less-advanced (Cluster 2). Additional tests, including Cohen’s *d* and Cohen’s U3, allowed us to measure the effect size—i.e., the magnitude of the difference between the means of more- and less-advanced regions in relation to the SNA measure applied.

### 3.3. Step 3—QCA

QCA explores causal relationships in complex systems and, more specifically, how multiple combinations of factors (also called configurations) can produce a given outcome [66]. In a nutshell, compared with regression analysis, the term “conditions” is used instead of independent variables, while the term “outcome” replaces dependent variable.

Regional development, path upgrading, innovation and engagement in research networks are all complex concepts that involve the interaction of several factors. Thus, QCA, which specifically addresses causal complexity, is well suited for the analysis.

In the case of csQCA (i.e., the specific type of QCA that we adopted), both the conditions and the outcome are expressed in a dichotomous form (i.e., (1) presence of the condition/outcome; (0) absence of the condition/outcome). In other words, after having identified a crossover point for the conditions and the outcome, it is possible to determine whether each case scores below or above the identified threshold [66, 67]. In our empirical analysis:

- The cases are represented by only the less-advanced EU regions grouped in Cluster 2;
- The conditions refer to the indicators previously listed in Sections 1 and 2 and described more accurately in Table 1: relative innovativeness, excellence in nanotechnology research, level of non-R&D innovation expenditure, size of the economy, quality of government, and resident population. The crossover point is the median of all of the variables adopted;
- A positive outcome corresponds to the ability shown by the less-advanced regions to score higher than the EU median in terms of degree centrality.

**Table 1:** QCA indicators: Variables, acronyms, descriptions, sources and years.

Indicator/Variable	Acronym	Description	Source	Year
Relative innovativeness	REL_INN	Ability shown by less-advanced regions to score higher than the national median in the Regional Innovation Scoreboard database. Such relative innovativeness seems to have a certain impact in determining a higher level of participation of the regions that we tagged as “less-advanced” [62]	Regional Innovation Scoreboard 2019	Various years preceding 2019
Excellence in nanotechnology research	NANO_EXCEL	Presence of at least one university in the Academic Ranking of World Universities (ARWU) with specific regard to the theme nanoscience and nanotechnology	ARWU	2017
Non-R&D innovation expenditure	NON_R&D	Investment in equipment, machinery and knowledge developed in other regions	EIS-RIS	2017
Quality of government	QoG	“*Citizens’ perceptions and experiences with corruption*, *quality and impartiality of three essential public services—health*, *education and policing—in their region of residence*” [68] based on the European Quality of Government Index	Regional Competitiveness Index 2019	2017
Size of economy	GDP	Regional GDP per capita	Eurostat	2017
Resident population	POP	Resident population	Eurostat	2017

In detail, the aim of our csQCA is to determine which combinations of conditions allow less-advanced regions to score higher than the EU median in terms of degree centrality in H2020-Nanotech, thereby greatly benefiting from multiscalar knowledge flows that may potentially trigger re-industrialization processes.

To achieve this goal, we created an overview of all the possible combinations of conditions and outcome set-memberships for the cases (i.e., a truth table). This allows a set-theoretic analysis of subset relationships—i.e., patterns of how conditions are combined for those who score highly in terms of network centrality [69]. In this sense, QCA sheds light on complex causal relationships. Moreover, the subset relationship analysis allows researchers to explore necessary and sufficient conditions. Conditions are necessary for those situations where, each time the outcome is present, the condition is also present. A sufficient condition produces the outcome, but is not necessary—i.e., other sufficient conditions can also produce the outcome. The necessary conditions must be present. However, this alone does not provide sufficiency; for the outcome to occur, it has to be in combination with the sufficient basis (the combinations, configurations, recipes) [66]. Given the necessary and sufficient conditions, QCA analyses the truth table and finds the minimal formula by means of a logical minimization process. The minimization is achieved by selecting and evaluating consistency and coverage [70], and in our case study, this was conducted using the fs/QCA software.

Consistency assesses the degree to which a subset relationship has been approximated, and coverage estimates how much of the sample the subset covers [71]. The recommended level for minimum consistency is 0.75 [66, 71, 72]. The results of our csQCA are presented in Section 4.3.

## 4. Empirical analysis

Through the combined statistical analyses and the three steps illustrated above, we have been able to determine the impact of the H2020-Nanotech in the less-advanced EU regions and its related possibility of fostering possible path upgrading [11]. We believe that our novel approach to the study of re-industrialization processes [8] and its related implications are beneficial for academics, policymakers and evaluators who are interested in determining the economic and, possibly, societal impact that the widespread adoption of a KET such as nanotechnology might have on long-term regional development dynamics and paths (e.g., [27]).

### 4.1. Cluster analysis—Results

**Fig 3:**
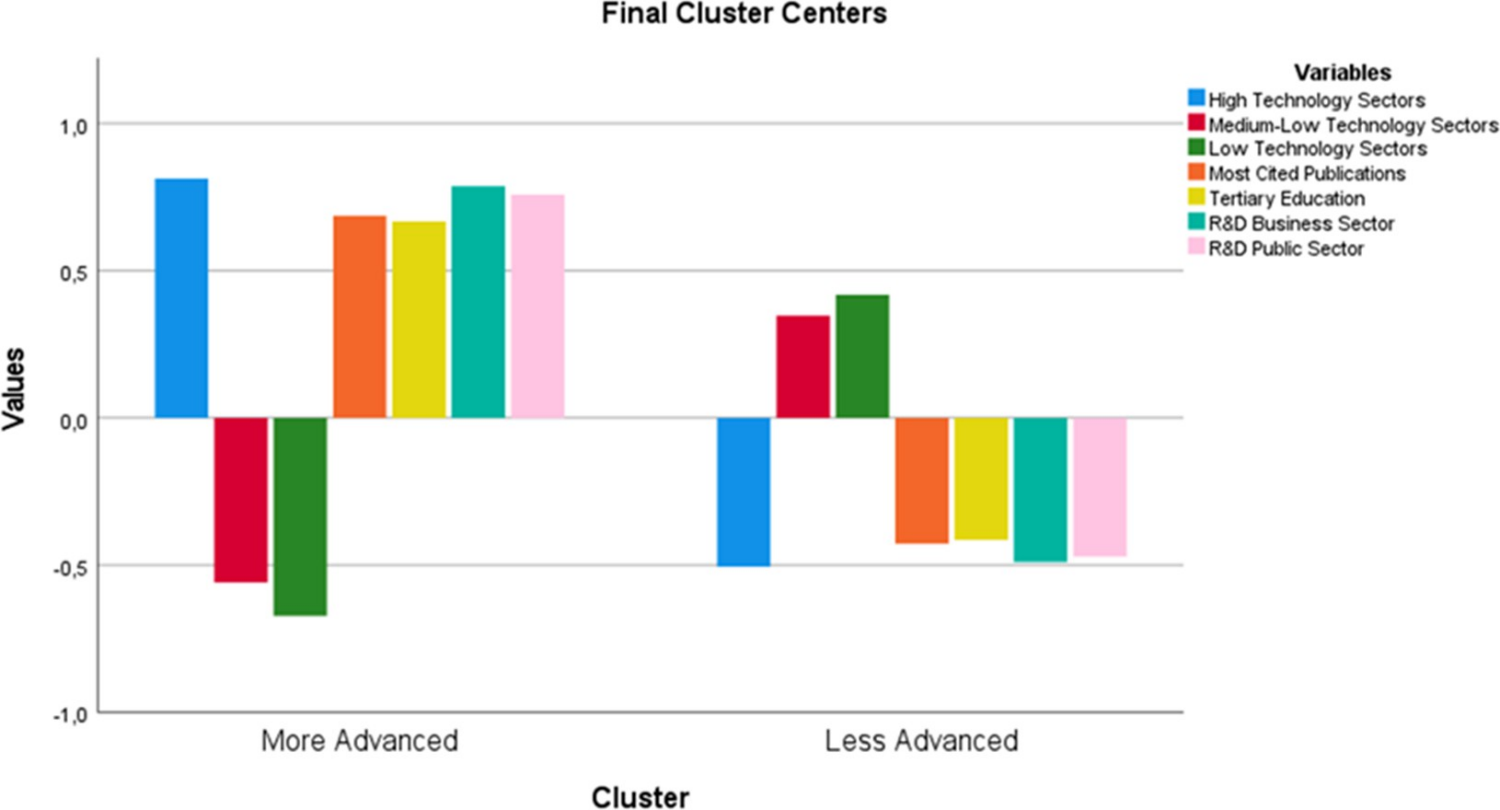
K-means clustering: Contribution of each variable to the formation of Cluster 1 (more-advanced regions) and Cluster 2 (less-advanced regions). Legend: Bars above zero—positive contribution; Bars below zero—marginal contribution; Length of the bars—the magnitude of the contribution.

We identified the more- and less-advanced EU regions by using K-means clustering. The bar chart in Fig 3 shows how the different variables contribute to the formation of each cluster. In particular, the bars above zero refer to the variables that contribute positively to the formation of the respective cluster, whereas, conversely, the bars below zero show the variables that contribute marginally. Finally, the length of the various bars refers to the importance of any such positive or negative contribution in each cluster identified.

As expected, the regions grouped in Cluster 1 (classified as more-advanced) are characterized by the presence of high-technology industries, scientific excellence, a considerable share of the population with higher education and R&D expenditure in the private and public sectors. In contrast, the regions making up Cluster 2 are characterized by a high proportion of medium- and low-technology industries, whereas scientific excellence, human capital (proxied by tertiary education) and R&D expenditure in both the private and public sectors play a very marginal role in the formation of this cluster. More specifically, the adoption of K-means clustering enabled us to identify 79 more-advanced regions and 127 less-advanced regions (see Table 2; for a complete list of both more- and less-advanced EU regions, see S3 Table).

**Table 2:** Number of regions in Cluster 1 (more advanced) and Cluster 2 (less advanced).

Cluster	Type of region	Quantity
1	More Advanced	79
2	Less Advanced	127
	Total	206

### 4.2. Network analysis and nonparametric tests—Results

The scores that are related to degree centrality, preliminarily, but clearly, show how the more-advanced EU regions (grouped in Cluster 1) play a central role in H2020-Nanotech.

The results reported in Table 3 show that a negligible number of less-developed EU regions (Cluster 2) are ranked among the top 25 regions. It is worth noting, in this regard, that the less-advanced regions that scored particularly highly in the EU nanotechnology network are exclusively located in Italy—i.e., a large and developed, but only moderately innovative, country, whose economic-production structure is still largely characterized by traditional sectors and small- and medium-sized manufacturing enterprises [73]. In addition to this, it should also be highlighted that these very few less-advanced regions that appear in the top 25 rankings represent some of the most developed and innovative areas in the moderately innovative Italian context (i.e., Lombardia, Emilia-Romagna, Piemonte and Veneto) and that they have traditionally performed well in the various editions of the EU FPs (concerning the importance of relative innovativeness in national contexts, see [62]; for comparison of previous rankings, see e.g., [52, 57]).

**Table 3:** Top 25 and 25 lowest-scoring regions. SNA measure: degree centrality.

Rank	Region	Country	Degree	Cluster	Rank	Region	Country	Degree	Cluster
1	Île de France	FR	150	1	182	Severozapaden	BG	0	*2*
2	Cataluña	ES	147	1	183	Yugoiztochen	BG	0	*2*
3	Oberbayern	DE	146	1	184	Yuzhen tsentralen	BG	0	*2*
4	Région de Bruxelles-Capitale	BE	144	1	185	Strední Cechy	CZ	0	*2*
5	Zuid-Holland	NL	144	1	186	Severozápad	CZ	0	*2*
6	Comunidad de Madrid	ES	143	1	187	Strední Morava	CZ	0	*2*
7	Lazio	IT	143	1	188	Kassel	DE	0	*2*
8	Vlaams Gewest	BE	141	1	189	Koblenz	DE	0	*2*
9	País Vasco	ES	138	1	190	Trier	DE	0	*2*
10	Attiki	EL	133	1	191	Peloponnisos	EL	0	*2*
11	Emilia-Romagna	IT	131	*2*	192	Notio Aigaio	EL	0	*2*
12	Lombardia	IT	131	*2*	193	Normandie	FR	0	*2*
13	Ostösterreich	DE	130	1	194	Molise	IT	0	*2*
14	Piemonte	IT	129	*2*	195	Nyugat-Dunántúl	HU	0	*2*
15	Helsinki-Uusimaa	FI	129	1	196	Zeeland	NL	0	*2*
16	Hovedstaden	DK	128	1	197	Zachodniopomorskie	PL	0	*2*
17	Auvergne—Rhône-Alpes	FR	127	1	198	Lubuskie	PL	0	*2*
18	Veneto	IT	127	*2*	199	Opolskie	PL	0	*2*
19	Eastern and Midland	IE	126	1	200	Podkarpackie	PL	0	*2*
20	South East	UK	126	1	201	Podlaskie	PL	0	*2*
21	London	UK	125	1	202	Centru	RO	0	*2*
22	Noord-Brabant	NL	123	1	203	Sud—Muntenia	RO	0	*2*
23	Västsverige	SE	123	1	204	Sud-Vest Oltenia	RO	0	*2*
24	Köln	DE	122	1	205	Vest	RO	0	*2*
25	Südösterreich	AT	122	1	206	Stredné Slovensko	SK	0	*2*

In Table 2, we also reported the 25 lowest-scoring regions. These regions are generally located in weakly or moderately innovative countries (i.e., Bulgaria, Czechia, Greece, Hungary, Italy, Poland, Romania and Slovakia), with some relevant exceptions (i.e., a few German, French and Dutch regions located in these more innovative countries). Despite these national differences, all of these 25 lowest-scoring regions belong to Cluster 2, which group the less-advanced EU regions, and are completely disconnected from the reconstructed nanotechnology network.

**Fig 4:**
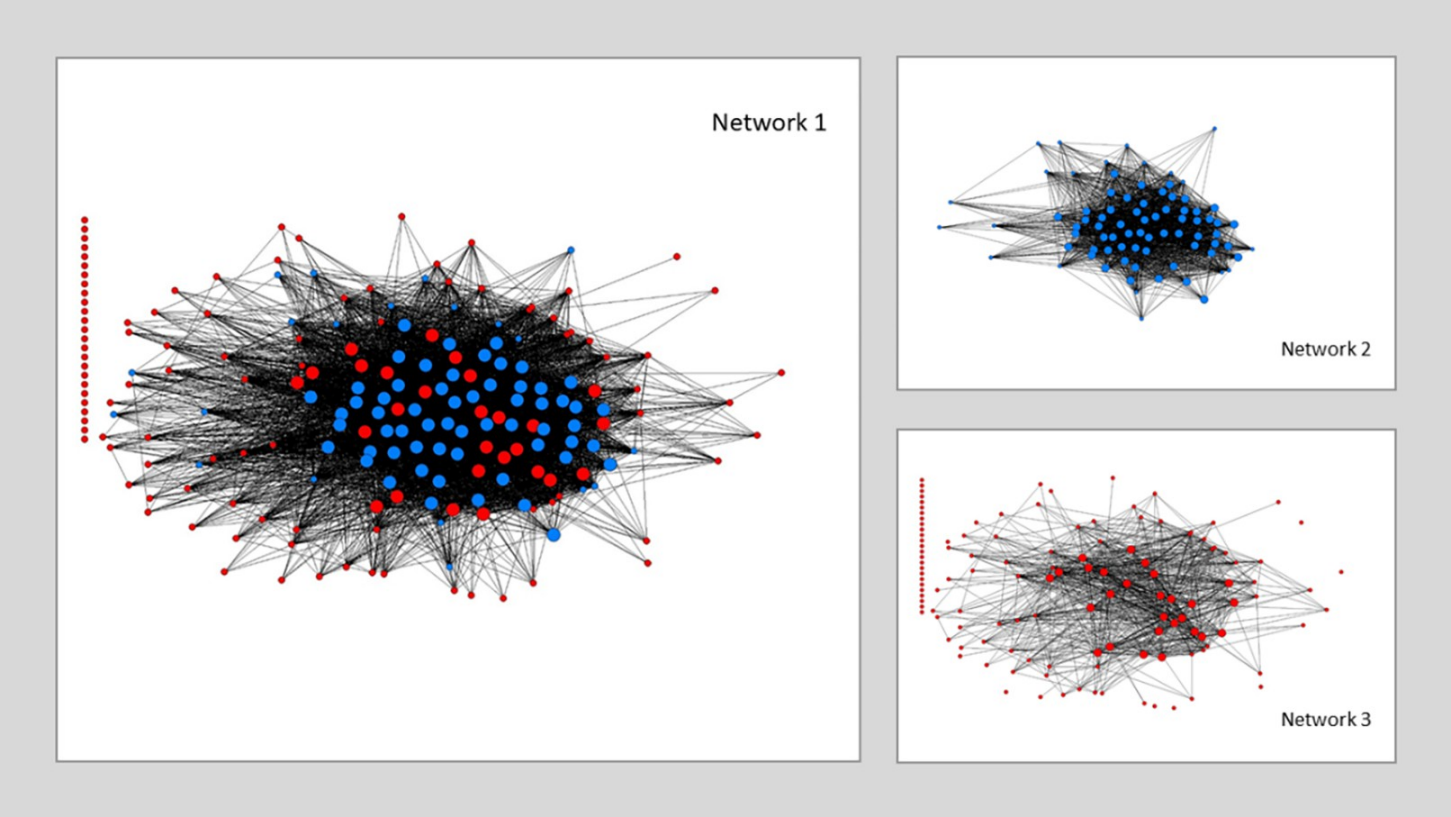
Core–periphery model (Ucinet elaboration; Borgatti et al., 2002 [74]). From top to bottom: Network 1—Complete H2020-Nanotech network (core: bigger circles; more-developed regions: blue circles; less-developed regions: red circles), Network 2—More-developed regions only (core: bigger circles), Network 3—Less-developed regions only (core: bigger circles). Core–periphery fit (correlation) = 0.90.

The predominance of the more-advanced EU regions in terms of centrality enables us to predict the existence of a core–periphery structure in H2020-Nanotech. This is confirmed by the high correlation fitness of the core–periphery model applied (0.90) and by the inspection of the three graphs presented in Fig 4.

In particular, the first graph shows all the connections established by each region in the FP subject to analysis. The blue circles represent the more-advanced regions (Cluster 1) and the red circles represent the less-advanced regions (Cluster 2), whereas the larger and more central circles highlight the core of the reconstructed network. The core is clearly dominated by the more-advanced regions, whereas the less-advanced regions are situated primarily in the peripheral part of the graph, or even disconnected completely, as shown by the large number of red circles with no connections placed in a column on the left. The subsequent graphs make the considerations related to the predominance of the more-advanced regions even clearer. The second graph refers solely to the more-developed regions and reveals how, in addition to being situated largely in the network core, the organizations located in these regions very often tend to collaborate with each other, thereby showing a high density within their reference group (0.74, which corresponds to 4,542 ties—i.e., 74% of connected regions). Conversely, the third graph shows that very few regions among the less-advanced regions make up the network core and are connected loosely with each other (i.e., the degree is 0.12, which corresponds to just 1,908 ties—i.e., 12% of connected regions). In this respect, a positive aspect is represented by the good number of connections between more- and less-developed regions, which is symptomatic of a moderate knowledge exchange between the two clusters. The densities and related number of ties within each group and between different groups (i.e., Density by Groups) are reported in Table 4.

**Table 4:** Densities and number of ties (in parentheses) within and between more-advanced regions (Cluster 1) and less-advanced regions (Cluster 2).

Density by groups		
	1	2
1	0.74 (4,542)	0.32 (3,236)
2	0.32 (3,236)	0.12 (1,908)

This result seems to confirm what was observed in previous studies—i.e., that more-advanced regions play a key or strategic role in the EU FPs, whereas less-advanced regions show a modest level of interaction and tend to create ties with the more-developed EU regions, which traditionally make up the core of the EU innovation networks throughout the various programming cycles. This peculiar dynamic seems to resemble preferential attachment—i.e., the tendency to create new ties with regions that already show a large number of connections (in this regard, see [56]).

After revealing some structural properties of the targeted network, we used nonparametric tests to validate the hypothesis that more-advanced EU regions perform considerably better than less-advanced regions in the H2020-Nanotech programme, thereby benefiting from more central positioning (degree centrality), which allows them to acquire new or complementary relevant knowledge. In particular, the adoption of the Mann–Whitney *U* test allowed us to demonstrate that the mean ranks of the more-advanced EU regions are considerably higher than their less-advanced counterparts (see Table 5 for details).

**Table 5:** Means, standard deviations and ranks (Mann–Whitney *U* test).

Variable	Cluster	No. of regions	Max.	Min.	Mean	Standard deviation	Mean rank	Sum of ranks
Degree	1	79	150	11	98.5	34.3	147.9	11,686
	2	127	131	0	40.5	40.9	75.9	9,635
	Total	206	150	0	62.7	47.8		

In addition to this, we used Cohen’s *d* test to provide some helpful information on the effect size (i.e., the relative strength of the differences between two means), and Cohen’s U3 test to show the percentage of scores in the cluster with the lower mean (Cluster 2) that are exceeded by the average score in the cluster with the higher mean (Cluster 1). Moreover, we performed an additional statistical analysis (probability of superiority) to present our results in an even more intelligible form.

**Fig 5:**
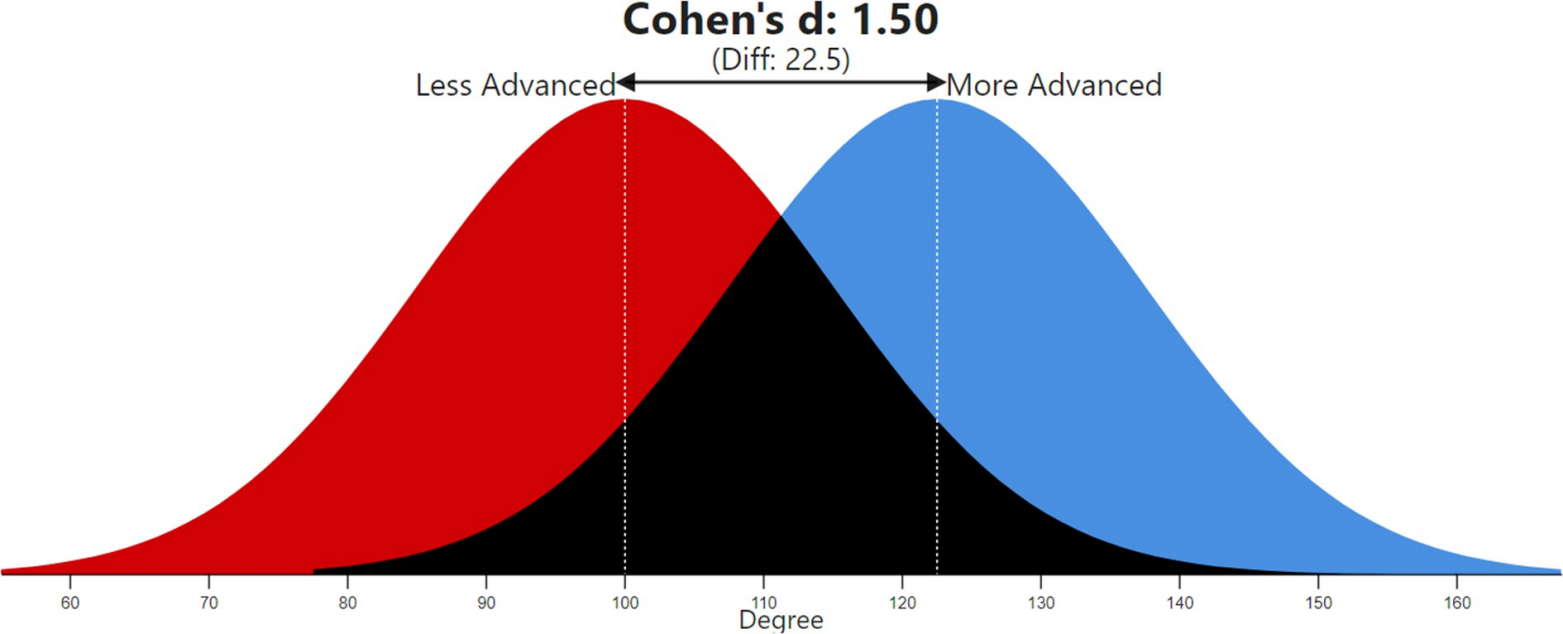
Cohen’s d effect size—Visualization (Magnusson, 2022) [75].

When calculating Cohen’s *d* score, our statistical analysis reveals a very large effect of 1.5 (scores > 0.8 imply a large effect). Moreover, the application of Cohen’s U3 test shows that more than 93% of the more-advanced regions (Cluster 1) score above the mean of the less-advanced regions (Cluster 2) in terms of degree centrality. This is further strengthened by the probability of superiority, which is a measure used to determine the chance that a more-developed region picked at random from Cluster 1 will have a higher score than a less-developed region picked at random from Cluster 2 [75]. The probability of superiority corresponds to 85.6% (see Table 6 for further details and Fig 5 for a graphical visualization of the statistical results).

**Table 6:** Nonparametric tests (Mann–Whitney *U*, Cohen’s *d*, Cohen’s U3, probability of superiority). * = Statistically significant at the .01 level.

	Variable—Degree
Test Statistics	
Mann–Whitney *U*	1.51
Wilcoxon *W*	9.6
*Z*	–8.4*
Cohen’s *d*	1.5*
Cohen’s U3	93.3%
Probability of superiority	85.6%
No. of regions	206

### 4.3. QCA—Results

In the final step, we conducted csQCA, whereby we investigated the combinations of conditions that enabled a few less-advanced regions to score higher than the EU median in the H2020-Nanotech programme, thus benefiting greatly from knowledge flows that potentially enabled the infusion of new technologies in the existing traditional sectors (i.e., possible re-industrialization processes).

As mentioned before, the conditions that we included in our QCA are relative innovativeness (REL_INN), excellence in nanotechnology research (NANO_EXCEL), level of non-R&D innovation expenditure (NON_R&D), size of the economy (GDP), quality of government (QoG) and resident population (POP) (see Table 1).

Based on the outcomes of our QCA, we were able to identify seven different combinations of conditions that allowed 33 of the 127 less-advanced regions to score higher than the EU median in terms of degree centrality. Our results show that a high level of participation depends primarily on scoring higher than the national median in the Regional Innovation Scoreboard (REL_INN) and in excellence in nanotechnology research (NANO_EXCEL). These two factors are present in almost all the combinations in which the degree centrality of the less-advanced regions is higher than the EU median (see the black circles displayed in Table 7, which show the combination in which a given condition has high importance). Similarly, high levels of GDP are needed to perform well in the EU nanotechnology network, whereas QoG and POP can be found in fewer combinations.

**Table 7:** QCA results. Legend: black circles (●) indicate a high level of a condition; empty circles (○) indicate a low level of a condition; blank cells indicate an irrelevant condition where the condition can be at a high or low level.

Outcome: DEGREE	REL_INN	NANO_EXCEL	NON_R&D	QoG	GDP	POP	Raw coverage	Unique coverage	Consistency
Combination 1	●	●			●	●	0.18	0.03	1
Combination 2	●		●		●	●	0.24	0.06	1
Combination 3		●		●	●	●	0.24	0.14	0.89
Combination 4	●	●	●		●		0.09	0.03	1
Combination 5	●	●	○	●	●		0.06	0.03	1
Combination 6	●	○	●	●		●	0.09	0.03	1
Combination 7	○	○	○	○	●	○	0.03	0.03	1

Solution coverage: 0.56

It is worth noting that there are always either high levels of REL_INN or NANO_EXCEL in the combinations we identified through csQCA, with the sole exception being combination 7, which seems to differ markedly from the patterns in combinations 1–6. QCA allows the identification of those cases (i.e., the less-advanced EU regions, in our analysis) that exemplify each combination. The most representative region in combination 7, where an exclusively high level of GDP was identified as a relevant condition, is Liguria (Italy). This is an industrialized and rich northern Italian region, which is conversely characterized by weaker research capacity compared with the rest of Europe, and scores lower than the median in terms of innovativeness in its respective national context (see [62]).

The overall solution consistency of the model we adopted is very high (i.e., 0.95), whereas the solution coverage (i.e., the extent to which a given outcome is explained by all of the observed configurations) is similarly well-accepted (i.e., 56% of the sample) [71].

To test the robustness of our QCA, we conducted a necessity test for both the original and the negated models [76]. A necessity test is used to determine whether a given condition can be considered sufficient to produce an outcome, although it must be clarified that the negated model refers to the absence of an outcome instead of its presence. In our case study, no single condition is necessary to produce the outcome (i.e., their consistency is constantly <0.9), although, interestingly, the negated model shows opposite results to the original model presented in Table 6. In particular, the negated model reveals the existence of eight combinations that lead to marginal participation in H2020-Nanotech (i.e., degree scores below the EU median). Remarkably, both low levels of REL_INN and NANO_EXCEL can be observed in six such identified combinations, whereas low levels of REL_INN were only found in a seventh combination. The results of the negated model are available upon request.

## 5. Discussion of the main results and concluding remarks

Although several previous studies have extensively discussed possible path upgrading in marginally innovative, peripheral or less-developed regions from a conceptual viewpoint or using a qualitative approach (e.g., [4, 11, 77, 78]), to our knowledge, this paper represents one of the very few attempts to quantify the possibility to trigger such a peculiar type of renewal in less-advanced regions.

In our research, we adopted a quantitative, transnational and multiscalar approach to determine the impact of the H2020-Nanotech programme (i.e., the most important innovation policy implemented by the EU) in terms of possible regional re-industrialization [8]. Our empirical analysis, which was conducted using various steps and a combination of several methods and techniques, was embedded in a broader theoretical background and achieved several, hopefully interesting, results.

The findings of this study, which was conducted at the EU level, strengthen what had previously been observed in a single country—i.e., that promoting major changes in an existing regional industrial path by the infusion of new technologies is definitely a desirable and even realistic solution for less-developed regions, although it appears to be difficult to achieve in practice (see [8]).

This is witnessed by an incontrovertible predominance of the more-advanced EU regions in terms of degree centrality, i.e., a result that is strengthened by the observation of a distinct core–periphery structure in H2020-Nanotech. This finding is consistent with what other scholars discovered in previously examined FPs (e.g., [51, 61]) and, in a sense, it seems to represent a sort of “wakeup call” for evaluators and policymakers because of the necessity of a more even and widespread participation between more- and less-developed regions, which has been highlighted repeatedly by both scholarly publications (in this regard, see e.g., [62]) and official documents and reports [79]. In this context, it is useful to mention again that previous studies have discovered the long-lasting existence of an elitist “club” of organizations and regions, which constitute a stable core that dominates the various EU FPs through its various programming cycles [51, 53, 61]—i.e., the “oligarchic networks” already identified by Breschi and Cusmano [60] two decades ago.

In this regard, the very marginal participation of less-developed regions in the EU nanotechnology network, and the related limited possibility to adopt nanotechnology solutions in low- and medium-technology industries, can be attributed to numerous factors. Although we did not directly determine the reasons that led to such a disappointing result, several previous studies conducted in a similar vein and in a similar context (i.e., the various EU programming cycles) provide us with possible explanations in this regard. For example, elements such as overlapping knowledge bases, absorptive capacity, and technological and cognitive proximity seem to shape collaborations within the EU FPs, by making interactions between organizations located in more-advanced regions easier and more frequent, while hindering the participation of organizations from less-developed regions [44, 80]).

Despite this, through the adoption of QCA, which is a novel method in the fields of economic geography and regional science, our study was able to reveal that a few less-advanced regions were able to play a central role in H2020-Nanotech, as well as the combinations of factors that allowed them to achieve such a positive result. Confirming the outcomes of a recent study conducted by Calignano [62], “relative innovativeness” (i.e., being innovation leaders in the respective national contexts, even in the case of marginally innovative countries) represents a key element in driving the participation of less-advanced regions. In addition to this, excellence in nanotechnology research and a comparatively high level of GDP per capita similarly lead to a satisfactory level of participation.

However, as can be inferred easily, the identified high-scoring regional areas represent a sort of “core within the periphery [in network terms]” (*authors’ note)* (see [81]) and are clear exceptions within a largely poorly equipped group of regions that are lagging behind. In other words, what we observe distinctly in the H2020-Nanotech programme is that a large majority of less-advanced regions (which can be defined as the “periphery of the periphery”; see, e.g., [82]) are only connected weakly to the rest of the network or are even completely isolated. These regions hardly benefit from critical multiscalar knowledge flows that have the potential to trigger an upgrade in the existing traditional medium-low- and low-technology industries.

**Fig 6:**
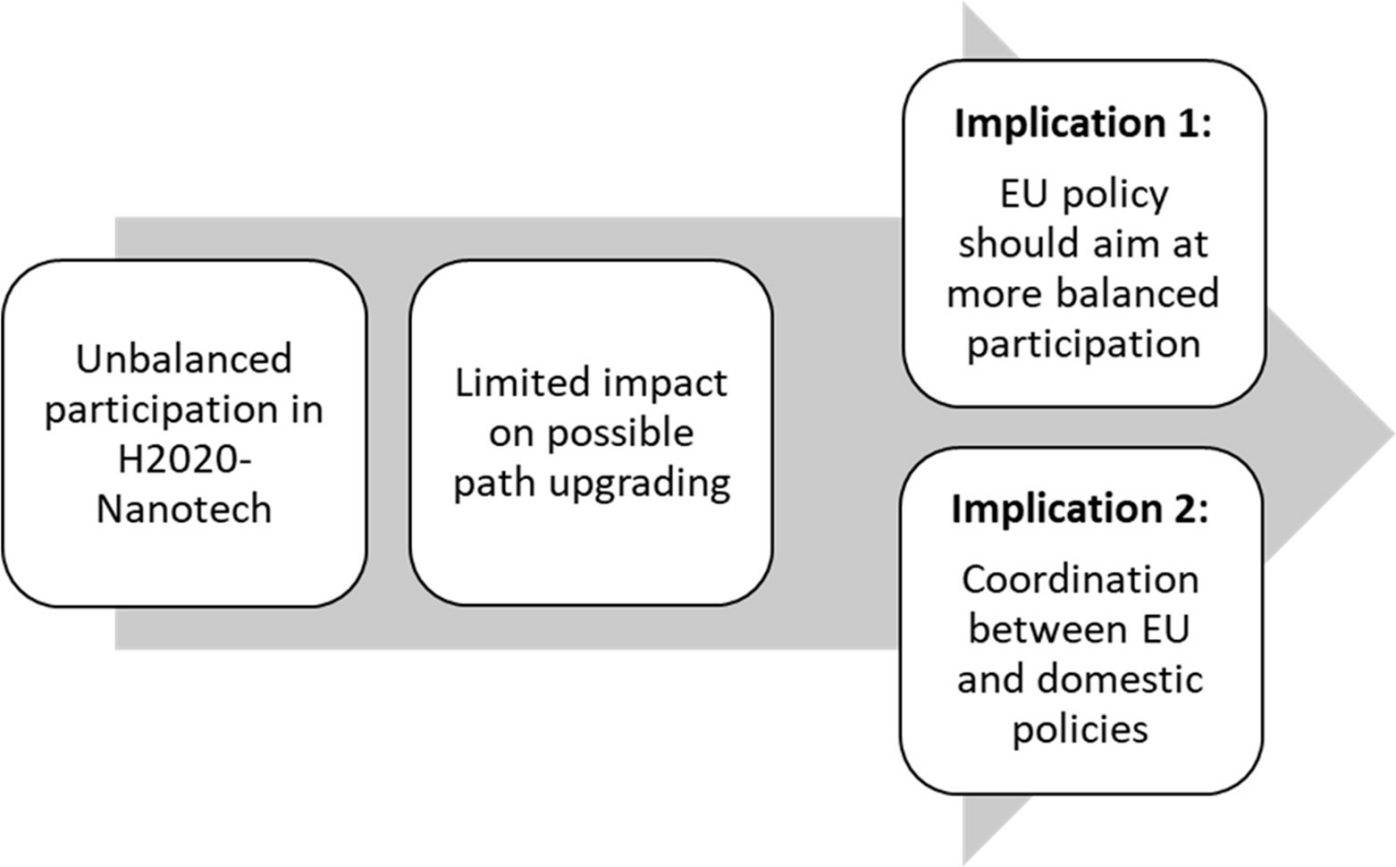
Research outcomes and policy implications.

The fact that less-advanced regions generally play a very marginal role in H2020-Nanotech not only has a limited impact on desirable re-industrialization processes (see also [8]) but also keeps the longstanding issue of more balanced participation in the FPs unresolved [44], despite the policy indications and specific policy actions implemented by the EU with the aim of supporting the strategies of the European Research Area (i.e., the inclusion of regions that lag behind in the virtuous circulation of resources, talent, knowledge and technologies) (see [79]). In this respect, as Calignano [62] stressed recently, it is necessary that the national governments and the EU evaluators and policymakers should work in closer and more effective cooperation in seeking to remove the structural barriers that prevent the satisfactory integration of a large number of lagging regions in the highly competitive research and innovation networks created within the various FPs. Among other things, the EU could start by strengthening its extant and not fully exploited policy measures (e.g., the aforementioned “Spreading Excellence and Widening Participation Programme”; [49]), while the various national governments could enhance their domestic knowledge bases and foster more widespread knowledge exchange through networking activities (for a broader discussion on the topic, see [62]; see Fig 6 for a graphical visualization of our research outcomes and policy implications).

Although we believe that the findings of the present empirical analysis have brought to light many interesting network dynamics and contribute positively to methodological and policy debates, it is necessary to acknowledge that our study is not exempt from limitations, First, it seems necessary to reiterate that the approach we used in this study allowed us to detect exclusively whether the EU nanotechnology network could potentially trigger path upgrading. Our research questions and empirical exercises are based on the theoretical assumption that transnational cooperation and the related infusion of relevant new technology (and knowledge) in predominant traditional sectors could engender this type of new development path in less-advanced regions (in this respect, see [3, 6, 8]). As a consequence, future studies could seek to understand whether, conversely, and in consideration of lacking EU funds and beneficial multiscalar knowledge flows, path upgrading may actually be and/or has been fostered by internal innovation and development dynamics (e.g., specific national policy and programs).

In extending our empirical approach, future research could identify the regions in which re-industrialization processes have taken place, and not study the possibility that this could happen, as in the present case study. This could be done by conducting before and after analyses on the structural features of the targeted regions (e.g., possible enhanced technological complexity, diversification of firms in related and unrelated sectors, higher shares of R&D personnel) and may help researchers to decipher which combinations of structural properties, socioeconomic conditions, and national and EU policy actions have led to the actual upgrading of the existing, less-advanced economic structures.

In addition to this, a critical aspect to consider is that the FPs may contribute to the success of the EU Cohesion Policy (i.e., more “harmonious development” between more- and less-developed EU countries and regions; [50]), but they do represent that policy themselves and may have a positive impact only indirectly and likely in combination with other structural measures. Hence, further analyses considering different sources of financial support, types of funding schemes and policy actions should be carried out to confirm what this paper seems to suggest, i.e., that path upgrading [11] or re-industrialization [8] have been activated in very few less-advanced EU regions possessing certain characteristics, but not in the large majority of them that remain largely excluded from beneficial multiscalar knowledge flows.

Another possibility that could be explored in the future is the link between different levels of participation in nanotechnology networks, new industrial processes and more sustainable development. Although this is beyond the scope of this paper, we have duly mentioned in our literature review the beneficial impact that nanotechnology might have on grand societal challenges such as more effective use of resources [14–16], climate change [17–19], health [20] and information and communication technology [21]. These theoretical and practical considerations, together with the availability of new data on environmental innovation issues provided by the latest version of the Community Innovation Survey (2018–2020), might lead to promising and still not fully developed research avenues.

## Supporting information

S1 Table Indicators, descriptions and databases. (DOCX)

S2 Table Iteration history (validation test)—Convergence reached after six iterations. (DOCX)

S3 Table List of more (Cluster 1) and less (Cluster 2) developed regions. Method: K-means clustering.
(DOCX)

S1 Fig Independent samples Mann–Whitney *U* test—Score distribution in more (Cluster 1) and less (Cluster 2) developed regions. SNA measures: degree.
(TIF)

## References

[1] Calignano G. Nanotechnology as a proxy to capture regional economic development? New findings from the European Union Framework Programmes, Nanotechnol Rev. 2017;6(2): 159–170.

[2] Butter M, Fischer N, Gijsbers G, Hartmann C, de Heide M., van der Zee MF. *Horizon 2020*: *Key Enabling Technologies (KETs)*, Booster for European Leadership in the Manufacturing Sector. Policy Department Economic and Scientific Policy–European Parliament, Brussels.

[3] Isaksen A, Trippl M. Path development in different regional innovation systems. In: Parrilli M., Fitjar RD, Rodríguez-Pose A, editors. Innovation Drivers and Regional Innovation Strategies. New York/Oxon: Routledge; 2016. Pp. 66–84.

[4] Asheim BT, Isaksen A, Trippl M. 2019. Advanced Introduction to Regional Innovation Systems. Edward Elgar.

[5] Asheim BT, Gertler MS. The geography of innovation: Regional innovation systems. In Fagerberg J, Mowery DC, Nelson RR, editors. The Oxford handbook of innovation. Oxford University Press; 2005. Pp. 291–317.

[6] Zukauskaite E., Trippl M., Plechero M. Institutional Thickness Revisited. Econ Geogr. 93:325–345.

[7] Blažek J., Květoň V. Towards an integrated framework of agency in regional development: the case of old industrial regions. Regional Studies. 2022 (forthcoming).

[8] Calignano G, Quarta CA. The Persistence of Regional Disparities in Italy Through the Lens of the EU Nanotechnology Network. Reg Stud Reg Sci. 2015;(2)1: 469–478.

[9] Chaminade C, de Fuentes C, Harirchi G, Plechero C. The geography and structure of Global Innovation Networks: global scope and regional embeddedness. In: Shearmur R, Carrincazeaux C, Doloreux D, editors. Handbook of the Geographies of Innovation; Cheltenham/Northampton: Edward Elgar; 2016. Pp. 370–381.

[10] Coe N, Bunnell T. Spatializing” knowledge communities: towards a conceptualization of transnational innovation networks. Glob Netw. 2003;3(4):437–456.

[11] Grillitsch M, Asheim BT, Trippl M. Unrelated knowledge combinations: the unexplored potential for regional industrial path development. Cambridge J Reg Econ Soc. 2018;11, 257–274.

[12] European Commission. Online Manual EU–Funding Programmes 2021–2027; 2022. https://ec.europa.eu/info/funding-tenders/opportunities/docs/2021-2027/common/guidance/om_en.pdf (Accessed 21.05.2023).

[13] European Commission. A new European research area based on excellence competitive, talent-driven and open; 2020. https://op.europa.eu/en/publication-detail/-/publication/aae418f1-06b3-11eb-a511-01aa75ed71a1/language-en (Accessed on 14.03.2022).

[14] Alvarado R., Tillaguango B., Dagar V., Ahmad M., Işık C., Méndez P., et al. Ecological footprint, economic complexity and natural resources rents in Latin America: Empirical evidence using quantile regressions. J Clean Prod. 2021;318:128585.

[15] Dagar V, Khan MK, Alvarado R, Usman M, Zakari A, Rehman A, et al. Variations in technical efficiency of farmers with distinct land size across agro-climatic zones: Evidence from India. J Clean Prod. 2021;315:128109.

[16] Manikandan S., Subbaiya R., Saravanan M., Ponraj M., Selvam M., Pugazhendhi A. A critical review of advanced nanotechnology and hybrid membrane based water recycling, reuse, and wastewater treatment processes. Chemosphere. 2022;289:132867. doi: 10.1016/j.chemosphere.2021.132867 34774910

[17] Ali M., Irfan M., Ozturk I., Rauf A. Modeling public acceptance of renewable energy deployment: a pathway towards green revolution. Econ Res-Ekon Istraz. 2023;36(3):2159849.

[18] Asif MA., Zhongfu T., Irfan M., Işık C. Do environmental knowledge and green trust matter for purchase intention of eco-friendly home appliances? An application of extended theory of planned behaviour. Environ Sci Pollut Res. 2023;30:37762–37774. 10.1007/s11356-022-24899-1 36574131

[19] Ramzan M, Abbasi KR, Salman A, Dagar V, Alvarado R, Kagzi M. Towards the dream of go green: An empirical importance of green innovation and financial depth for environmental neutrality in world’s top 10 greenest economies. Technol Forecast Soc Change. 2023;189:122370.

[20] Chintapula U., Chikate T., Sahoo D., Kieu A., Guerrero Rodriguez ID., Nguyen KT., et al. Wiley Interdiscip Rev Nanomed Nanobiotechnol. 2023;15(1):e1840. 35950266 10.1002/wnan.1840 PMC9840662

[21] Zhang C, Khan I, Dagar V, Saeed A, Zafar MW. Environmental impact of information and communication technology: Unveiling the role of education in developing countries. Technol Forecast Soc Change. 2022;178:121570.

[22] Pokrajac L, Abbas L, Chrzanowski W, Dias GM, Eggleton BJ, Maguire S, et al. Nanotechnology for a Sustainable Future: Addressing Global Challenges with the International Network 4 Sustainable Nanotechnology. ACS Nano. 2021;15(12):18608–18623. doi: 10.1021/acsnano.1c10919 34910476

[23] Wanzenböck I, Wesseling JH, Frenken K, Hekkert MP, Weber KM. A framework for mission-oriented innovation policy: Alternative pathways through the problem–solution space. Sci Public Policy. 2020;47(4):474–489.

[24] Kuhlmann S., Rip A. Next-Generation Innovation Policy and Grand Challenges, Sci Public Policy. 2018:45(4): 448–454.

[25] Pilkington B. The Current State of Nanotechnology in Europe. Azo Nano. 2022. Available from: https://www.azonano.com/article.aspx?ArticleID=6203.

[26] International Standard Organizations, 2005. ISO/TC 229 –Nanotechnologies. https://www.iso.org/committee/381983.html (Accessed on 12.03.2022).

[27] Roco MC. The long view of nanotechnology development: the National Nanotechnology Initiative at 10 years. J Nanopart Res.2011;13:427–445.

[28] Graffenberger M. Bypassing Structural Shortcomings: Innovative Firms in Peripheral Regions. In: Lang T, Görmar F, editors. Regional and Local Development in Times of Polarisation, New Geographies of Europe. Palgrave Macmillan; 2019. Pp. 287–317.

[29] Rypestøl JO., Aarstad J. Entrepreneurial innovativeness and growth ambitions in thick vs. thin regional innovation systems. Entrepr Region Dev. 2018;30:639–661.

[30] Grabher G. The weakness of strong ties: the lock-in of regional development in the Ruhr-area. In: Grabher G, editor. The Embedded Firm: on the Socioeconomics of Industrial Networks. London: Routledge; 1993. Pp. 255–278.

[31] Hassink R. How to unlock regional economies from path dependency? From learning region to learning cluster, Eur Plan Stud. 2005;13:521–535.

[32] Trippl M, Otto A. How to turn the fate of old industrial areas: a comparison of cluster-based renewal processes in Styria and the Saarland. Environ Plan A; 41:1217–1233.

[33] Morgan K. Nurturing novelty: regional innovation policy in the age of smart specialisation. Environ Plan C. 2017;35:569–583.

[34] Schulze-Krogh AC., Calignano G. How do firms perceive interactions with researchers in small innovation projects? Advantages and barriers for satisfactory collaborations. J Knowl Econ. 2020;11,3: 908–930.

[35] Bathelt H, Glückler J. 2011. The relational economy. Geographies of knowing and learning. Oxford University Press.

[36] Dai S, Taube M, Liu J, Liu G. Innovation Network Formation and the Catalyzing State: A Study of Two Innovative Industry Clusters in China. Journal Contemp China. 2023 (*forthcoming*).

[37] Van Egeraat C, Kogler D, Cooke P. Global and regional dynamics in knowledge flows and innovation, editors. New York/London: Routledge; 2014.

[38] Powell WW, Grodal S. Networks of innovators. In: Fagerberg J., Mowery DC, Nelson RR, editors. The Oxford Handbook of Innovation. Oxford: Oxford University Press; 2005. Pp. 56–85.

[39] Aslesen HW, Harirchi G. 2015. The effect of local and global linkages on the innovativeness in ICT SMEs: does location-specific context matter?. Entrepr Region Dev;2015;27:644–669.

[40] Bathelt H, Turi P. Local, global and virtual buzz: The importance of face-to-face contact in economic interaction and possibilities to go beyond. 2011. Geoforum;42:520–529.

[41] Torre A. On the role played by temporary geographical proximity in knowledge transmission. Reg Stud. 2008;42:869–889.

[42] Aarstad J, Kvitastein OA, Jakobsen S-E. Local buzz, global pipelines, or simply too much buzz? A critical study. Geoforum. 2016;75:129–133.

[43] Fitjar RD, Rodríguez-Pose A. Innovating in the periphery: Firms, values, and innovation in southwest Norway. Eur Plan Stud. 2011;19:555–574.

[44] Amoroso S, Coad A, Grassano N. European R&D networks: a snapshot from the 7^th^ EU Framework Programmes. Econ Innov New Technol. 2018;27:404–419.

[45] Guffarth D, Barber MJ. The evolution of aerospace R&D collaboration networks on the European, national and regional levels. In: Vermeulen B, Paier M, editors. Innovation networks for regional development. Concepts, case studies, and agent-based models. Springer; 2017. Pp. 15–50.

[46] Meliciani V, Di Cagno D, Fabrizi A, Marini M. Knowledge networks in joint research projects, innovation and economic growth across European regions. Ann Reg Sci. 2021 (forthcoming).

[47] Balland PA, Boschma R, Ravet J. Network dynamics in collaborative research in the. EU, 2003–2017, Eur Plan Stud. 2019;27(9):1811–1837.

[48] Olechnicka A, Ploszaj A, Celińska-Janowicz D. The geography of scientific collaborations. London/New York: Routledge; 2019.

[49] European Commission. Spreading Excellence & Widening Participation in Horizon 2020. Analysis of FP participation patterns and research and innovation performance of eligible countries; 2018. https://ec.europa.eu/info/research-and-innovation/funding/funding-opportunities/funding-programmes-and-open-calls/horizon-2020_en (Accessed on 14.03.2022).

[50] European Commission. New cohesion policy; 2021. https://op.europa.eu/en/publication-detail/-/publication/151f4fdc-2c97-11ec-bd8e-01aa75ed71a1 (Accessed on 14.03.2022).

[51] Balland PA, Suire R, Vicente J. Structural and geographical patterns of knowledge networks in emerging technological standards: Evidence from the European GNSS industry. Econ Innov and New Technol. 2013;22:47–72.

[52] Calignano G, Trippl M. Innovation-driven or challenge-driven participation in international energy innovation networks? Empirical evidence from the H2020 programme. Sustainability. 2020;12(11): 4696.

[53] Roediger-Schluga T., Barber MJ. R&D collaboration networks in the European Framework Programmes: Data processing, network construction and selected results. Int J Foresight Innov Policy. 2008;4:321–347.

[54] Hoekman J, Scherngell T, Frenken K, Tijssen R. Acquisition of European research funds and its effect on international scientific collaboration. J Econ Geog. 2013;13:23–52.

[55] Wanzenböck I., Scherngell T., Lata R. Embeddedness of European regions in European Union-funded research and development (R&D) networks: A spatial econometric perspective. Reg Stud. 2015;49:1685–1705.

[56] Calignano G, Fitjar RD, Hjertvikrem N. Innovation networks and green restructuring: Which path development can EU Framework Programmes stimulate in Norway?. Nor Geogr Tidsskr. 2019;73(1): 65–78.

[57] Calvo-Gallardo E, Arranz N., Fernández de Arroyabe JC. Analysis of the European energy Innovation system: Contribution of the Framework Programmes to the EU policy objectives. J Clean Prod. 2021;298:126699.

[58] Calvo-Gallardo E, Arranz N, Fernandez de Arroyabe C. Contribution of the Horizon 2020 Program to the Research and Innovation Strategies for Smart Specialization in Coal Regions in Transition: The Spanish Case. Sustainability. 2022;14(4):2065.

[59] Di Cagno D, Fabrizi A, Meliciani V, Wanzenböck I. The impact of relational spillovers from joint research projects on knowledge creation across European regions. Technol Forecast Soc Change. 2016;108:83–94.

[60] Breschi S, Cusmano L. Unveiling the texture of a European Research Area: emergence of oligarchic networks under EU Framework Programmes. International Journal of Technology Management. 2004:27:747–772.

[61] Enger SG. Closed clubs: Network centrality and participation in Horizon 2020. SciPublic Policy. 2018;45:884–896.

[62] Calignano G. Not all peripheries are the same: The importance of relative regional innovativeness in transnational innovation networks. Growth Change. 2022;53(1): 276–312.

[63] Calignano G, Nilsen T, Jørgensen Nordli A, Hauge A. Beyond ‘Periphery’: A detailed and nuanced taxonomy of the Norwegian regions, Geogr Ann Ser B (f*orthcoming*).

[64] Hanneman R. A., & Riddle M. (2005). Introduction to social network methods. University of California.

[65] Borgatti SP, Everett GM. Models of core/periphery structures. Soc Netw. 2000;21:375–395.

[66] Ragin CC. Fuzzy-set social science. Chicago: University of Chicago Press; 2000.

[67] Schneider CQ., Wagemann C. Standards of good practice in qualitative comparative analysis (QCA) and fuzzy-sets. Comp Sociol. 2010;9:397–418.

[68] Charron N, Lapuente V, Annoni P. Measuring quality of government in EU regions across space and time. Papers in Regional Science. 2019;98(5):1925–1953.

[69] Ragin CC, Strand SI, Rubinson C (2008). User’s guide to fuzzy-set/qualitative comparative analysis. University of Arizona, 87, 1–87.

[70] Longest K.C., Vaisey V. Fuzzy: A program for performing qualitative comparative analyses (QCA) in Stata. Stata J. 2008;8:79–104.

[71] Ragin CC. Set relations in social research: Evaluating their consistency and coverage. Polit Anal. 2006;14:291–310.

[72] Fiss PC. Building better causal theories: A fuzzy set approach to typologies in organization research. Acad Manage J. 2011;54:393–420.

[73] Bellandi M, Lombardi S, Santini E. Traditional manufacturing areas and the emergence of product-service systems: the case of Italy. Journal of Industrial and Business Economics. 2020;47:311–331.

[74] Borgatti SP, Everett MG, Freeman LG. Ucinet 6 for Windows: Software for Social Network Analysis. Analytic Technologies. 2002.

[75] Magnusson K. (2022). Interpreting Cohen’s d effect size: An interactive visualization (Version 2.5.2) *[Web App]*. R Psychologist. https://rpsychologist.com/cohend/ (Accessed on 30.08.2022).

[76] Skaaning SE. Assessing the robustness of crisp-set and fuzzy-set QCA results. Sociol Methods Res. 2011;40:391–408.

[77] Grillitsch M, Asheim BT. Place-based innovation policy for industrial diversification in regions. Eur Plan Stud. 2018;26:1638–1662.

[78] Jolly S, Grillitsch M, Hansen T. Agency and actors in regional industrial path development. A framework and longitudinal analysis. Geoforum. 2020;111:176–188.

[79] European Parliament. Overcoming innovation gaps in the EU-13 Member States; 2018. https://www.europarl.europa.eu/RegData/etudes/STUD/2018/614537/EPRS_STU(2018)614537_EN.pdf (Accessed on 14.03.2022).

[80] Scherngell T, Barber MJ. Distinct spatial characteristics of industrial and public research collaborations: evidence from the fifth EU Framework Programme. Ann Reg Sci. 2011;46:247–266.

[81] Pugh R, Dubois A. Peripheries within economic geography: Four “problems” and the road ahead of us. J Rural Stud. 2021;87:267–285.

[82] Glückler J, Shearmur R, Martinus K. Liability or opportunity? Reconceptualizing the periphery and its role in innovation. J Econ Geog. 2023; 23(1):231–249.

