# Supplementary material for: Less-advanced regions in EU innovation networks: Could nanotechnology represent a possible trigger for path upgrading?
Source: PLoS One. 2024 Jan 12;19(1):e0288669. doi: 10.1371/journal.pone.0288669 (PMC10786367; doi:10.1371/journal.pone.0288669)
Supplement: S1 Table — (DOCX) [file pone.0288669.s001.docx]

**Supporting information**

**S1 Table.**

| **Indicator** | **Description** | **Database** |
| --- | --- | --- |
| High-technology sectors | Average share of total employment in high-technology sectors (e.g., pharmaceutical, computer, electronics, optical products, aircraft and spacecraft). Years: 2013–2019 | Eurostat |
| Medium-low-technology sectors | Average share of total employment in medium-low-technology sectors (e.g., petroleum products, rubber and plastic products, ships and boats, and repair and installation of machinery and equipment). Years: 2013–2019 | Eurostat |
| Low-technology sectors | Average share of total employment in low-technology sectors (e.g., food and beverages, tobacco products, textiles, wood products and furniture). Years: 2013–2019 | Eurostat |
| Scientific excellence | Scientific publications among the top 10% most cited publications worldwide as a percentage of total scientific publications of the respective country | Regional Innovation Scoreboard 2019 |
| Human capital | Share of population with tertiary education | Regional Innovation Scoreboard 2019 |
| R&D intensity/1 | R&D expenditure in the public sector as a percentage of GDP | Regional Innovation Scoreboard 2019 |
| R&D intensity/2 | R&D expenditure in the private sector as a percentage of GDP | Regional Innovation Scoreboard 2019 |

**S4 Table.**

| **Cluster 1—More-developed regions (79)** |
| --- |
| **Belgium:** Région de Bruxelles-Capitale / Brussels Hoofdstedelijk Gewes, Vlaams Gewest, Région Wallonne **Czechia:** Praha **Denmark:** Hovedstaden, Sjælland, Midtjylland, Nordjylland **Germany:** Stuttgart, Karlsruhe Freiburg, Tübingen, Oberbayern, Oberpfalz, Mittelfranken, Berlin, Brandenburg, Bremen, Hamburg, Darmstadt, Gießen, Braunschweig, Hannover, Köln, Rheinhessen-Pfalz, Chemnitz, Leipzig **Ireland:** Northern and Western, Southern, Eastern and Midland **Greece:** Attiki **Spain:** País Vasco; Comunidad de Madrid; Cataluña **France:** Île de France, Bretagne, Languedoc-Roussillon—Midi-Pyrénées, Auvergne—Rhône-Alpes; Provence—Alpes-Côte d'Azur **Italy:** Lazio **Hungary:** Budapest **Lithuania:** Sostinės regionas **Netherlands:** Groningen, Drenthe, Overijssel, Gelderland, Flevoland, Utrecht, Noord-Holland, Zuid-Holland, Noord-Brabant, Limburg **Austria:** Ostösterreich Südösterreich **Poland:** Warszawski stoleczny **Portugal:** Lisboa **Romania:** Bucuresti-Ilfov **Slovenia:** Zahodna Slovenija **Slovakia:** Bratislavský kraj **Finland:** Helsinki-Uusimaa Etelä-Suomi Länsi-Suomi Pohjois- ja Itä-Suomi **Sweden:** Stockholm, Östra Mellansverige, Sydsverige, Västsverige; Övre Norrland **United Kingdom:** North East, North West, East Midlands, West Midlands, East of England, London, South East, South West, Wales, Scotland, Northern Ireland |
| **Cluster 1—Less-developed regions (127)** |
| **Bulgaria:** Severozapaden, Severen tsentralen, Severoiztochen, Yugoiztochen, Yugozapaden, Yuzhen tsentralen **Czechia:** Strední Cechy, Jihozápad, Severozápad, Severovýchod, Jihovýchod, Strední Morava, Moravskoslezsko **Denmark:** Syddanmark **Germany:** Niederbayern, Oberfranken, Unterfranken, Schwaben, Kassel, Mecklenburg-Vorpommern, Lüneburg, Weser-Ems, Düsseldorf, Münster, Detmold, Arnsberg, Koblenz, Trier, Saarland, Dresden, Sachsen-Anhalt, Schleswig-Holstein, Thüringen **Greece:** Anatoliki Makedonia, Thraki, Kentriki Makedonia, Thessalia, Dytiki Ellada, Sterea Ellada, Peloponnisos, Notio Aigaio, Kriti **Spain:** Galicia, Principado de Asturias, Cantabria, Comunidad Foral de Navarra, La Rioja, Aragón, Castilla y León, Castilla-la Mancha, Extremadura, Comunidad Valenciana, Illes Balears, Andalucía, Región de Murcia **France:** Centre-Val de Loire, Bourgogne—Franche-Comté, Normandie, Nord-Pas de Calais—Picardie, Alsace—Champagne-Ardenne—Lorraine, Pays de la Loire, Aquitaine—Limousin—Poitou-Charentes **Croatia:** Jadranska Hrvatska, Kontinentalna Hrvatska **Italy:** Piemonte, Liguria, Lombardia, Provincia Autonoma Bolzano/Bozen, Provincia Autonoma Trento, Veneto, Friuli-Venezia Giulia, Emilia-Romagna, Toscana, Umbria, Marche, Abruzzo, Molise, Campania, Puglia, Basilicata, Calabria, Sicilia, Sardegna **Hungary:** Pest, Közép-Dunántúl, Nyugat-Dunántúl Dél-Dunántúl, Észak-Magyarország, Észak-Alföld, Dél-Alföld **Lithuania:** Lietuvos regionas **Netherlands:** Friesland, Zeeland **Austria:** Westösterreich **Poland:** Malopolskie, Slaskie, Wielkopolskie, Zachodniopomorskie, Lubuskie, Dolnoslaskie, Opolskie, Kujawsko-Pomorskie, Warminsko-Mazurskie, Pomorskie, Lódzkie, Swietokrzyskie, Lubelskie, Podkarpackie, Podlaskie, Mazowiecki regionalny **Portugal:** Norte, Centro, Alentejo **Romania:** Nord-Vest, Centru, Nord-Est, Sud-Est, Sud—Muntenia, Sud-Vest Oltenia, Vest **Slovenia:** Vzhodna Slovenija **Slovakia:** Západné Slovensko, Stredné Slovensko, Východné Slovensko **Sweden:** Småland med öarna, Norra Mellansverige, Mellersta Norrland **United Kingdom:** Yorkshire and The Humber |
