# Supplementary material for: Less-advanced regions in EU innovation networks: Could nanotechnology represent a possible trigger for path upgrading?
Source: PLoS One. 2024 Jan 12;19(1):e0288669. doi: 10.1371/journal.pone.0288669 (PMC10786367; doi:10.1371/journal.pone.0288669)
Supplement: S2 Table — (DOCX) [file pone.0288669.s002.docx]

**S2 Table.**

| Iteration History | | |
| --- | --- | --- |
| Iteration | Change in Cluster Centers | |
| Iteration | **1** | **2** |
| 1 | 3.765 | 3.673 |
| 2 | 0.054 | 0.034 |
| 3 | 0.031 | 0.018 |
| 4 | 0.057 | 0.034 |
| 5 | 0.048 | 0.031 |
| 6 | 0 | 0 |
