# Supplementary figures and images for: Less-advanced regions in EU innovation networks: Could nanotechnology represent a possible trigger for path upgrading?
Source: PLoS One. 2024 Jan 12;19(1):e0288669. doi: 10.1371/journal.pone.0288669 (PMC10786367; doi:10.1371/journal.pone.0288669)

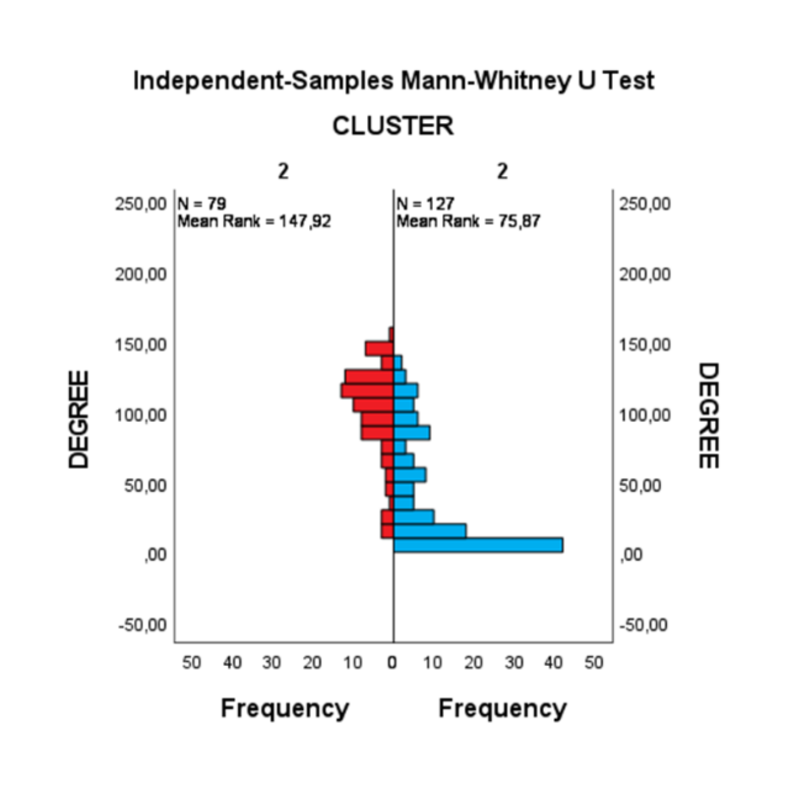

Supplement: S1 Fig — SNA measures: degree. (TIF) [file pone.0288669.s004.tif]
